# Gene expression of enzymes involved in utilization of xylooligosaccharides by *Lactobacillus* strains

**DOI:** 10.1080/13102818.2014.948257

**Published:** 2014-10-30

**Authors:** Ananieva Maria, Tzenova Margarita, Iliev IIlia, Ivanova Iskra

**Affiliations:** ^a^ Faculty of Biology, Sofia University, Sofia,Bulgaria; ^b^ Faculty of Biology, Plovdiv University, Plovdiv, Bulgaria

**Keywords:** gene expression, *Lactobacillus*, xylooligosaccharides, XOS

## Abstract

Prebiotics are defined as food components that confer health benefits on the host through modulation of the microbiota. Xylooligosaccharides (XOS) are non-digestible oligosaccharides that have recently received increasing attention as potential prebiotic candidates. XOS are sugar oligomers composed of 1,4-linked xylopyranosyl backbone and are obtained by either chemical or, more commonly, enzymatic hydrolysis of xylan polysaccharides, extracted from the plant cell wall. The bifidogenic effect of XOS was demonstrated by both in vitro studies and small-scale in vivo human studies. Some intestinal bacterial strains are able to grow on XOS, yet numerous studies have demonstrated that the ability to utilize these oligosaccharides varies considerably among these bacteria. The aim of this study is to investigate the ability of several strains *Lactobacillus* to use XOS. Fifteen *Lactobacillus* strains, allifiated to *L. plantarum*, *L. brevis* and *L. sakei*, were studied. Screening procedure was performed for the ability of the strains to utilize XOS as an alternative carbon source. Only some of them utilize XOS. The growth kinetics show the presence of two lag phases, indicating that these bacteria utilize probably some monosaccharides present in the used XOS. XOS were fermented with high specificity by *Bifidobacteria* strains, but *Lactobacilli* did not metabolize XOS efficiently.

## Introduction

Xylooligosaccharides (XOS) are sugar oligomers made up of xylose units, which appear naturally in bamboo shoots, fruits, vegetables, milk and honey. Their production at an industrial scale is carried out from lignocellulosic materials. XOS can be used for several purposes. Prebiotics are commonly non-digestible oligosaccharides which can improve the composition of the gut microbiota eliciting beneficial health effects. With a growing market for prebiotic-containing foods, there is an increasing interest in understanding how prebiotics function at a molecular level. Two approved prebiotics are fructooligosaccharides (FOS) and galactooligosaccharides (GOS), while β-(1→4) linked XOS with degree of polymerization (DP) of 2–10 are considered emerging prebiotics. Similar to the approved prebiotics, FOS and GOS, XOS enhance growth of probiotic *Bifidobacterium* and *Lactobacillus* species [1] and pathogens, e.g. *Clostridium*. XOS are neither hydrolysed nor absorbed in the upper part of the human gastrointestinal tract. They affect the host by selectively stimulating the growth of limited number of beneficial bacteria such as *Bifidobacteria* and *Lactobacillus*. The importance of XOS as a valuable food ingredient is increasing in the present scenario as it possess a variety of health benefiting effects such as lowering the cholesterol, improving the biological availability of calcium, etc. Moreover, it has acceptable organoleptic property and does not exhibit toxicity or negative effects on human health. They also exhibit a wide range of biologic activities including antioxidant activity, antimicrobial, anti-allergy, anti-infection, anti-inflammatory properties, selective cytotoxic activity, immunomodulatory action, cosmetic and various other properties.

High amount of XOS production is usually accomplished by xylanases with less or negligible amount of exo-xylanase or β-xylosidase activity as the presence of exo-xylanase or β-xylosidase activity produces high amount of xylose which causes an inhibitory effect on the production of XOS.[2]

The aim of the present work was to determine the growth rate and antimicrobial activity of *Lactobacillus* strains cultivated in a media containing 2% XOS. The expression of the genes of two enzymes involved in the degradation of XOS was also investigated.

## Materials and methods

### Bacterial growth, growth conditions and RNA extraction

#### Bacterial strains

In this study 15 strains from Lactic acid bacteria isolated from Bulgarian national meat products (Lukanka) were used. All the strains were identified using 16S RNA techniques. In this study 13 strains *Lactobacillus plantarum* (S1, S2, S3, S4, S5, S6, S18, S20, S33, S38, S35, S37 and S40) and 2 strains *Lactobacillus brevis* (S8 and S27) were used.

Origin of XOS – xylooligosaccharides – 95% Powder (Longlive Bio-technology Co., Ltd, Chine) – concentration – not supported and MRS broth (Merck Germany) 20 g/L glucose concentration.

#### Fermentation and dynamic of bacterial growth

Overnight cultures of the studied strains were washed twice with 0.85% NaCl solution. 200 μL from the bacterial suspension were used to inoculate modified MRS (de Man, Rogosa and Sharpe) broth medium (pH 6.8), containing 2% XOS.[3,4]

The control consisted of the same amount of 200 μL from the bacterial suspension, used for the inoculation with MRS broth medium.

Bacterial growth was measured by a turbidimetric method at 600 nm and calibrated against MRS broth using spectrophotometer (Biochrom Libra). The OD readings and standard deviations were calculated using duplicate samples from two separate experiments.[5]

RNA was extracted with GeneMATRIX Universal RNA Purification Kit from an overnight culture according to manufacturer instructions (EURx Poland).

#### Primers and primer design

The multiplex primers used in this study were designed using GeXP express Profiler software (Beckman Coulter) and are given as follows:

*For *β*-xylosidase*

Forward primer: GTGACACTATAGAATACGCGCAACATCTACAATGGG;

Reverse primer: GTACGACTCACTATAGGGACCAACGTACTCCAACCACGA.

*For xylanase*

Forward primer: AGGTGACACTATAGAATA CGCGCTTTGCATGCTATTCT;

Reverse primer: GTACGACTCACTATAGGGA AAATCCAGATGGCGAACGGT.

*For glucose-6-phosphate 1-dehydrogenase*

Forward primer: AGGTGACACTATAGAATA TCTTTGGCGGTACTGGTGAC;

Reverse primer: GTACGACTCACTATAGGGA TGTTGGGCAATCGTACCGAA including universal tags. Concentration of each primer was 10 pmol/μL per reaction.

Each primer contained universal tags (according to Beckman Coulter protocol), the reverse primer contained a 19-nucleotide universal priming sequence at the 5′ end and the forward primer consisted of an 18-nucleotide universal priming sequence at the 5′ end. Inside the GeXP PCR buffer there were two pairs of primers labelled with D4 dye which were complementary to the 5′ end of the designed primers. All the PCR products were designed >3-bp apart, ranging from 60 to150 bp, so that they could be distinguished by capillary electrophoresis analyses. In addition to the two genes of interest, each panel contained one housekeeping gene and Kanamycin RNA (Kan RNA) – additional not supported information that served as an external control. Kan RNA can produce a peak at 325 bp after capillary electrophoresis. The concentrations of all chemicals were not supported by the producer.

#### Reverse transcription

The reaction volume was 20 μL (containing 4 μL of 5 × RT buffers, 5 μL KanR RNA, 2 μL reverse primer, 1 μL reverse transcriptase enzyme (from GeXP Starter kit, Beckman Coulter, USA) and 5 μL RNA). In addition to the primer and Taq enzyme, other reagents were supported by a GenomeLab GeXP Start Kit (Beckman Coulter). The RT reaction was done in a thermal cycler (VWR-Doppio) under the following conditions: 1 min at 48 °C, 60 min at 42 °C, 5 min at 95 °C, and hold at 4 °C. Each experiment included a RT-Minus control and a no-template control (NTC) to ensure each peak provided the expected result. The RT-Minus control was a no-enzyme reaction to ascertain if the RNA was contaminated with DNA. The NTC was a no-template control to confirm all that reaction reagents were in good condition. The concentrations of all chemicals were not supported by the producer.

#### Polymerase chain reaction (PCR)

The reaction volume was 12 μL (4 μL of 5 × RT buffers, 4 μL 25 mM MgCl2, 0.7 μL of Thermo-Start DNA Polymerase and 2.0 μL of forward primer plex) and 9.30 μL of cDNA samples from the RT plate. In addition to the primers, other reagents were supported by Beckman Coulter. The PCR reaction was done in a thermal cycler (VWR-Doppio) under the conditions: 10 min at 95 °C, followed by 40 cycles of 30 s at 94 °C, 30 s at 55 °C and 1 min at 68 °C, and hold at 4 °C.

#### GeXP fragment and data analyses

The procedure for GeXP fragment and data analyses followed the manufacturer instructions. To the PCR products from the multiplex reaction were added l μL to the appropriate wells of a new 96-well sample microplate. A total of 0.5 μL DNA size standard 400 (GenomeLab GeXP Start Kit; Beckman Coulter) was added to 38.5 μL of sample loading solution with thorough mixing. The mix solution was assembled and added to the 96-well sample microplate. The PCR product was separated based on fragment size by capillary gel electrophoresis (GeXP Genetic analysis system, Beckman Coulter USA). The strength of the dye signal was measured after normalization to KanR RNA. Normalization of the signals were performed in GeXP express profiler software (Beckman Coulter, USA)

#### Antimicrobial activity

Antimicrobial assay was performed by the well diffusion method [5,6]. After adjusting the pH to 6.5 by NaOH, the activity of the collected samples (100 μL) was checked against test organisms belonging to different species as *Escherichia coli 3398*, *Enterobacter 3690* and *Staphylococcus aureus 746*. Test cultures were cultivated on nutrition broth and agar. The plates were incubated overnight at 30 and 37 °C.

## Results and discussion

Screening procedure was performed for the ability of the strains to utilize 2% XOS in mMRS as an alternative carbon source.

The received data indicate that 13 strains *L. plantarum* used XOS. However, the variation between the strains is significant. Comparable consumers of XOS are *L. plantarum* strains S1, S6, S35 and *L. brevis* strain S38. Those strains showed good ability to utilize XOS as an alternative carbon source. Other investigated strains showed less ability to utilize XOS.[7]

The screening of *Lactobacilli* showed that the utilization of XOS of all tested species *L. plantarum* was variable. Only *L. plantarum* S1 used XOS efficiently as an alternative carbon source. For the other strains, the growth with XOS was about 20%–25% of growth supported by glucose.

The two strains *L. brevis* used XOS similar to the strains of *L. plantarum* S1 and S6.

The experimental data of the growth rate are comparable with the data of pH change. The highest acidification was observed from strains *L. plantarum* S1, S40 and *L. brevis* S8 and S27.

**Figure 1.**
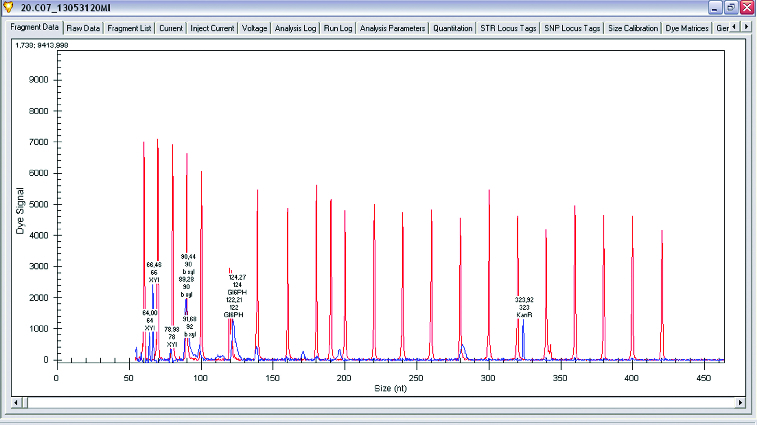
Gene expression levels of xylanase, β-xylosidase and glucose-6-phosphate dehydrogenase in *Lactobacillus plantarum*.

**Figure 2.**
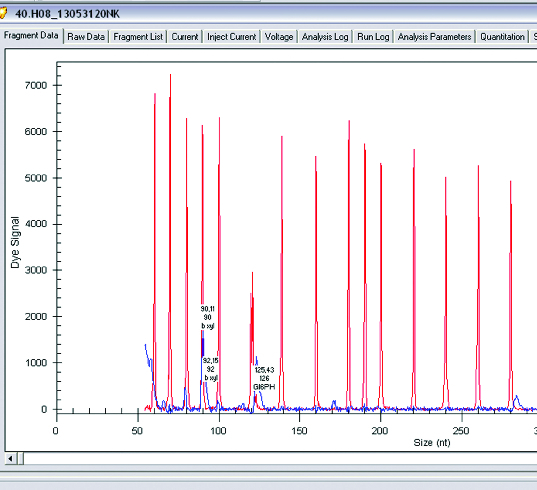
Gene expression levels of β-xylosidase and glucose-6-phosphate dehydrogenase in *Lactobacillus plantarum*.

**Figure 3.**
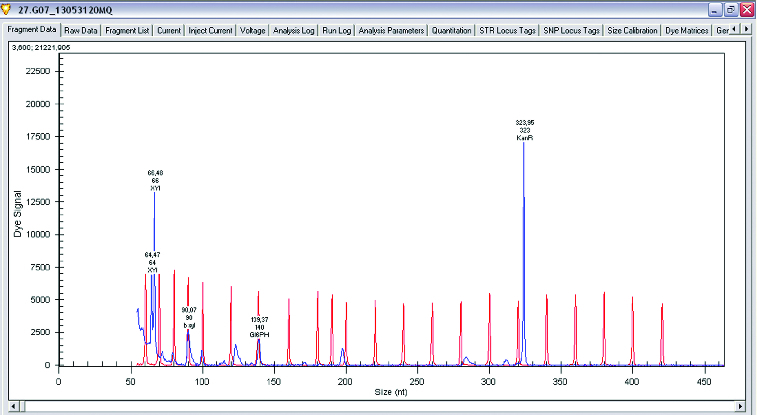
Gene expression levels of xylanase, β-xylosidase and glucose-6-phosphate dehydrogenase in *Lactobacillus brevis*.

**Figure 4.**
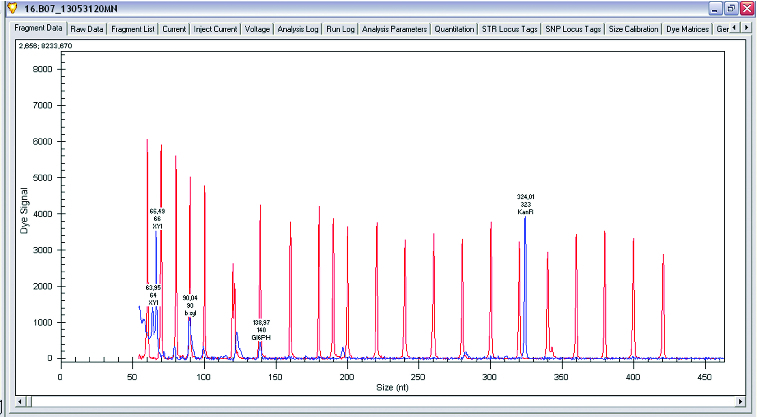
Gene expression levels of xylanase, β-xylosidase and glucose-6-phosphate dehydrogenase in *Lactobacillus sakei*.

The main enzymes involved in the utilization of XOS are β-xylosidase and xylanase. A research of gene expression of two enzymes involved in degradation of XOS xylanase and β-xylosidase was performed (*L. plantarum* S1, S20 and S40, *L. brevis* S27). Semi-quantitative analysis of expression levels of β-xylosidase and xylanase was done on GenomeLab GeXP system. To normalize levels of expression, *glu6* and KanR were used as housekeeping genes (*glu6* – gene for glucose-6-phosphate dehydrogenase and internal control KanR gene coding Kanamycin from GeXP starter kit for gene expression). The performed analysis showed that when *Lactobacillus* strains were growing on XOS, the genes for xylanase (which degrade the linear polysaccharide beta-1,4-xylan into xylose) and glucose-6-phosphate dehydrogenase are upregulated. Some of the strains show expression only of xylanase and only few of them showed expression of both enzymes xylanase and β-xylosidase. Analysis of gene expression of the enzymes of interest showed that xylanase is upregulated when strains are grown on XOS. Some strains of *L. plantarum* (S1, S20 and S40) expressed two enzymes of interest in higher levels, but some of them have expressed only β-xylosidase (Figures 1–4).

The examined two *L. brevis* strains expressed xylanase and β-xylosidase and also had a gene for glucose-6-phosphate dehydrogenase.

**Figure 5.**
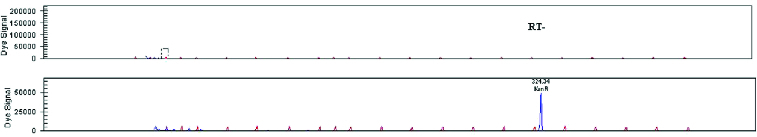
RT-Minus control and no-template control (NTC).

For conformation of the presence of each pick in the samples, RT-Minus control and NTC were used. They showed no expression of any genes of interest which ensured that each peak provided the expected result (Figure 5).

Levels of expression of xylanase and β-xylosidase against glucose-6-phosphate dehydrogenase and KanR gene are shown in Table 2.

**Table 1.** Optical density and pH changing of investigated strains.

	Optical density 600 nm on 24 hour		Changing the pH on 24 hour	
Strain	Control 2% glucose	2% XOS	Control 2% glucose	2% XOS
*L. plantarum S1*	1.709	0.990	4.02	3.51
*L. plantarum S2*	0.632	0.450	4.12	4.53
*L. plantarum S3*	0.903	0.583	3.88	4.53
*L. plantarum S4*	0.976	0.440	3.95	3.76
*L. plantarum S5*	0.900	0.490	3.91	4.41
*L. plantarum S6*	1.350	0.600	3.84	3.85
*L. plantarum S18*	1.292	0.430	5.04	4.46
*L. plantarum S20*	0.820	0.630	4.39	4.92
*L. plantarum S33*	0.888	0.810	4.01	4.62
*L. plantarum S35*	0.440	0.710	5.55	4.43
*L. plantarum S37*	1.180	0.685	4.00	4.67
*L. plantarum S38*	1.190	0.844	5.39	4.84
*L. plantarum S40*	0.916	0.662	4.75	3.86
*L. brevis S8*	1.801	1.013	5.21	3.01
*L. brevis S27*	1.350	0.845	5.40	3.50

**Table 2.** Levels of gene expression of enzymes of interest in *Lactobacillus plantarum* and *Lactobacillus brevis*.

	Gene expression level			
Strain	Xylanase	β-xylosidase	glucose-6-phosphate dehydrogenase	KanR
*L. plantarum*	++	+++	+	+
*L. brevis*	+++	++	+	+++
*L. sakei*	+++	++	+	++

Note:

‘+’ – weak expression (less than 1000 units).

‘++’ – medium expression (between 1000 and 3000 units).

‘+++’ – higher expression (more than 3000 units).

### Antimicrobial activity

**Figure 6.**
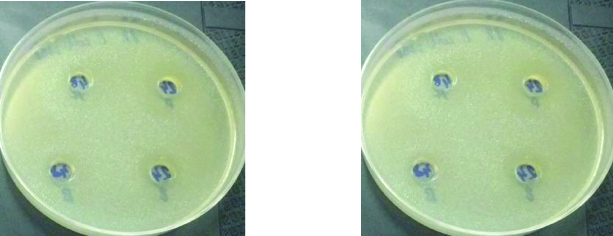
Antimicrobial activity of *L. plantarum* and *L. brevis* (2) against *E. coli 3398* on 24th and 48th hour 1 – *L. plantarum* 2 – *L. brevis*.

**Figure 7.**
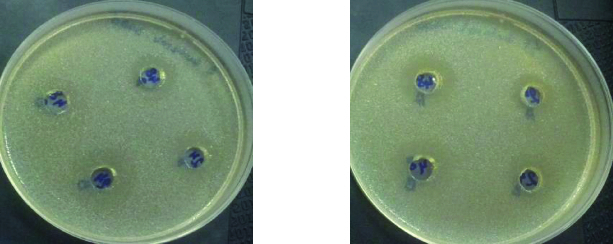
Antimicrobial activity of *L. plantarum* (1) and *L. brevis* (2) against *Enterobacter 3690* on 24th and 48th hour 1 – *L. plantarum* 2 – *L. brevis*.

**Figure 8.**
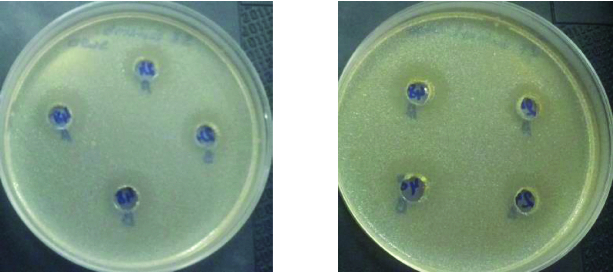
Antimicrobial activity of *L. plantarum* (1) and *L. brevis* (2) against *Staphylococcus aureus 746* on 24th and 48th hour 1 – *L. plantarum* 2 – *L. brevis*.

All the examined strains had not antimicrobial activity when cultivated on glucose (data not shown). After cultivation on XOS some of the strains showed good antimicrobial activity against several test organisms (*E. coli 3398*, *Enterobacter 3690 Staphylococcus aureus 746*). The obtained results are shown in Table 3 and Figures 6–8.

**Table 3.** Antimicrobial activity spectrum of the cell-free supernatant of strains *Lactobacillus plantarum* and *Lactobacillus brevis* grown on 2% XOS.

	Antimicrobial activity/mm sterile zone/														
Indicator strains	*Lactobacillus plantarum*													*Lactobacillus brevis*	
	*S1*	*S2*	*S3*	*S4*	*S5*	*S6*	*S18*	*S20*	*S33*	*S35*	*S37*	*S38*	*S40*	*S8*	*S27*
*E. coli 3398*	22	20	18	22	24	25	23	22	24	26	25	21	20	24	0
*Enterobacter 3690*	28	25	23	25	26	25	23	26	26	25	22	23	24	27	25
*Staphylococcus aureus 746*	28	22	24	26	23	24	24	22	20	21	21	22	22	27	25

It is very interesting to note that all the studied strains when cultivated on XOS had antimicrobial activity, which does not differ significantly when the three test cultures were used. It could be noted that the induction of activity in the presence of XOS was higher especially for *L. plantarum* S1 and *L. brevis* S8.

The observed antimicrobial activity against some food pathogens after cultivation on XOS also indicated that the system of uptake of unusual sugars induced a specific way of production of antimicrobial substances. The mechanism of this stimulation remains unclear.[8]

## Conclusion

We consider present work to be the preliminary study of the utilization of XOS and gene expression of enzymes xylanase and β-xylosidase by the *Lactobacillus* strains isolated from meat products. Utilization of XOS by Lactic acid bacteria is strain specific. The obtained results may help to explain the ability of *Lactobacillus* strains to compete with other bacteria in the ecosystem of the human gastro-intestinal tract.
